# PPM1G promotes the progression of hepatocellular carcinoma via phosphorylation regulation of alternative splicing protein SRSF3

**DOI:** 10.1038/s41419-021-04013-y

**Published:** 2021-07-21

**Authors:** Dawei Chen, Zhenguo Zhao, Lu Chen, Qinghua Li, Jixue Zou, Shuanghai Liu

**Affiliations:** 1 Department of Hepatopancreatobiliary Surgery, Jiangyin People’s Hospital, School of Medicine, Southeast University, No. 163, Shoushan Road, Jiangyin, 214400 Jiangsu Province China; 2 Liver Cancer Institute, Zhongshan Hospital, Fudan University, No. 180, Fenglin Road, Shanghai, 200032 China

**Keywords:** Oncogenes, Mechanisms of disease, Prognostic markers, Liver cancer

## Abstract

Emerging evidence has demonstrated that alternative splicing has a vital role in regulating protein function, but how alternative splicing factors can be regulated remains unclear. We showed that the PPM1G, a protein phosphatase, regulated the phosphorylation of SRSF3 in hepatocellular carcinoma (HCC) and contributed to the proliferation, invasion, and metastasis of HCC. PPM1G was highly expressed in HCC tissues compared to adjacent normal tissues, and higher levels of PPM1G were observed in adverse staged HCCs. The higher levels of PPM1G were highly correlated with poor prognosis, which was further validated in the TCGA cohort. The knockdown of PPM1G inhibited the cell growth and invasion of HCC cell lines. Further studies showed that the knockdown of PPM1G inhibited tumor growth in vivo. The mechanistic analysis showed that the PPM1G interacted with proteins related to alternative splicing, including SRSF3. Overexpression of PPM1G promoted the dephosphorylation of SRSF3 and changed the alternative splicing patterns of genes related to the cell cycle, the transcriptional regulation in HCC cells. In addition, we also demonstrated that the promoter of PPM1G was activated by multiple transcription factors and co-activators, including MYC/MAX and EP300, MED1, and ELF1. Our study highlighted the essential role of PPM1G in HCC and shed new light on unveiling the regulation of alternative splicing in malignant transformation.

## Introduction

Hepatocellular carcinoma (HCC) is one of the most aggressive malignant tumors worldwide. Owing to rapid tumor growth and easy metastasis, <30% of patients with HCC can be treated by surgery [1–3]. In addition, the recurrence rate of HCC is over 40% after therapy [4]. Therefore, identifying the key molecular markers of HCC and exploring its mechanism is the key to clinically improving the overall treatment effect of HCC and reducing its mortality.

Emerging evidence has shown that alternative splicing plays a vital role in protein function [5–10]. Typically, many biological elements are involved in the process—from DNA to proteins [11–13]. First, the DNA is transcribed to pre-RNAs using many types of transcriptional regulatory machinery. Secondly, the RNAs are spliced into mRNA in the RNA splicing machinery. Then, the spliced RNAs are exported from the nucleus to the cytoplasm and translated into proteins in the translational machinery. The same pre-RNA can be spliced at different splicing sites to produce different mRNA isoforms; thus, alternative splicing significantly increases the diversity of proteins. Recently, many researchers have shown that alternative splicing plays an essential role in malignancies, including HCC [7–10]. For instance, the alternative splicing events of the androgen receptor (AR)—the driver oncogene in prostate cancer—resulted in an AR-V7 isoform [14]. The AR-V7 is specifically highly expressed in patients with relapse and drug resistance after targeted therapy.

RNA splicing is regulated by various splicing factors, including the serine/arginine-rich (SR) protein family [15]. The SR proteins contain the RNA recognition motif (RRM) and the arginine/serine-rich (RS) domain. The RRM can recognize and bind to sequence the specific-RNA motif, whereas the RS domain exerts the splicing function. Splicing factors require distinct post-translational modifications, mostly phosphorylation or dephosphorylation, to exert the splicing function. The phosphorylase and dephosphorylase are key to the splicing activity of SR proteins. We analyzed a panel of phosphorylase and dephosphorylase in HCC. Among these proteins, PPM1G (Protein phosphatase, Mg2+/Mn2+ dependent 1 G) was overexpressed and related to adverse risk in HCC (Fig. S1). PPM1G is an important member of the PP2C family of serine/threonine protein phosphatases and has an important role in controlling cell cycle progression [16, 17]. Several studies have reported that the PPM1G might be involved in the dephosphorylation of pre-mRNA splicing factors [18, 19], but the detailed targets of PPM1G and its function in malignancies remain largely unexplored.

Here, we investigated the role of PPM1G in HCC. We explored the clinical relevance of PPM1G in HCC and showed the high levels of HCC were related to advanced HCC stages and poor prognosis. We analyzed the function of PPM1G in HCC both in vitro and in vivo and showed that the knockdown of PPM1G inhibited cell growth and the invasion of HCC. Moreover, we performed co-immunoprecipitation (Co-IP) and RNA-seq and showed that the PPM1G regulated the alternative splicing of genes related to cell cycle and transcriptional regulation via the dephosphorylation of SRSF3. Finally, we analyzed the upstream regulators of PPM1G and found that the PPM1G was activated by MYC/MAX and EP300, MED1, ELF1, ETV4.

## Results

### PPM1G was highly expressed in HCC

**Fig. 1:**
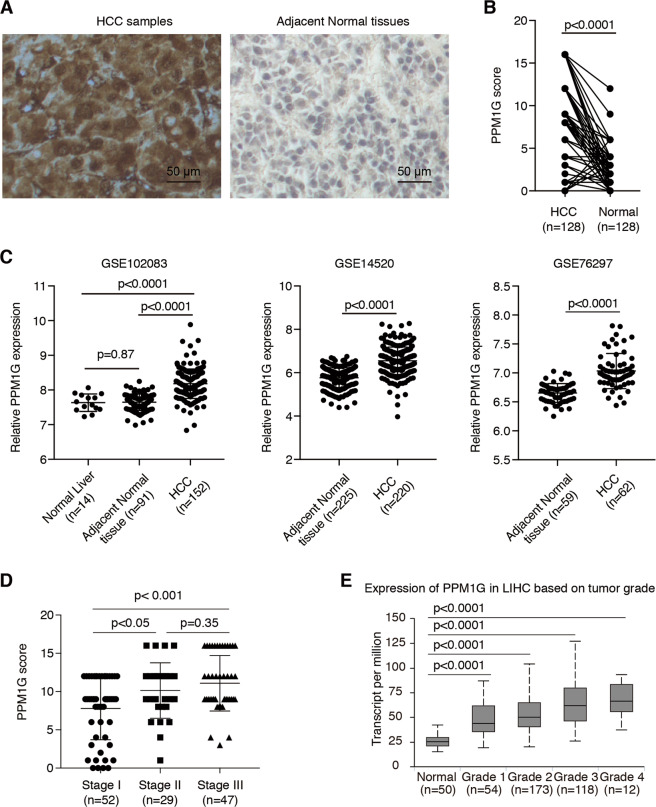
PPM1G was highly expressed in HCC cells. **A**–**B** PPM1G was highly expressed in the HCC samples as compared with adjacent normal tissues. The IHC experiments were performed in HCC samples and adjacent normal tissues using antibodies against PPM1G (**A**). The statistical comparison of the PPM1G levels between HCC samples and adjacent normal tissues was shown (**B**). **C** The mRNA levels of PPM1G were significantly higher in HCC tissues than adjacent normal tissues or normal liver tissues in the GEO database. **D** The levels of PPM1G were increased in the advanced stages of HCC. The PPM1G scores in the different stages of HCC were shown. **E** The mRNA levels of PPM1G were highly expressed in HCC. The levels of PPM1G in the TCGA-LIHC cohort were analyzed.

To investigate the expression of PPM1G in HCC, we first analyzed the expression of PPM1G in HCC samples and adjacent normal tissues by immunohistochemistry (IHC). We found that the expression of PPM1G in HCC samples was much higher than those in their adjacent normal tissues (Fig. 1A), suggesting the PPM1G might be related to the malignancies of HCC. Statistical analyses showed that the PPM1G expression was significantly higher in HCC tissues than in normal adjacent tissues (Fig. 1B). Also, we retrieved the expression profiling in the GEO database and analyzed the expression of PPM1G in these cohorts. Consistent with the IHC staining results, the mRNA levels of PPM1G were significantly higher in HCC tissues than adjacent normal tissues or normal liver tissues (Fig. 1C).

Next, we analyzed the expression of PPM1G in different stages of HCC and found that the expression of PPM1G was much higher in stage III HCC than stage II and stage I HCC (Fig. 1D), suggesting the expression of PPM1G might increase in an advanced stage. Similarly, we retrieved the expression of PPM1G in the tumor samples and the normal samples in the TCGA-LIHC cohort, and the results in TCGA-LIHC supported the findings in our cohort (Fig. 1E).

### High levels of PPM1G were correlated with a poor prognosis of HCC

**Fig. 2:**
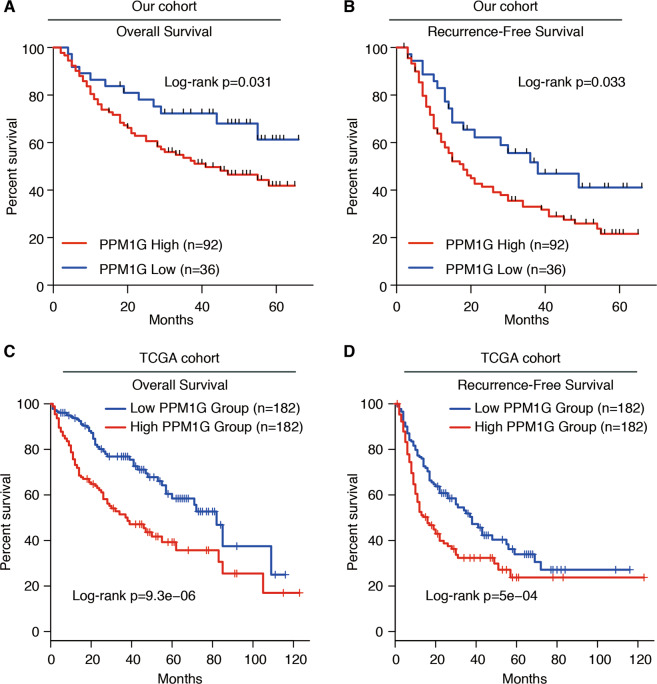
High levels of PPM1G were related to the poor prognosis of HCC. **A** Kaplan–Meier analysis of the overall survival in PPM1G-high and PPM1G-low groups of our HCC cohort. **B** Kaplan–Meier analysis of the recurrence-free survival in PPM1G-high and PPM1G-low groups of our HCC cohort. The expression of PPM1G was divided into two groups according to the PPM1G score. **C** Kaplan–Meier analysis of the overall survival in PPM1G-high and PPM1G-low groups of the TCGA-LIHC cohort. **D** Kaplan–Meier analysis of the recurrence-free survival in PPM1G-high and PPM1G-low groups of the TCGA-LIHC cohort. The expression of PPM1G was divided into two groups according to the PPM1G mRNA expression.

Next, we analyzed the correlation between PPM1G and overall survival/recurrence-free survival in HCC. We divided the HCC cases into two groups according to the expression of PPM1G. The overall survival rate in the group with high PPM1G expression was lower than the group with relative PPM1G-low expression (Fig. 2A). Similarly, high PPM1G levels showed a poor prognosis in recurrence-free survival (Fig. 2B). These observations were also supported by the TCGA-LIHC cohort (Fig. 2C–D). Together, these observations indicated that the higher levels of PPM1G were a poor prognostic factor for HCC.

### Knockdown PPM1G inhibited the cell growth and invasion in HepG2 and Hep3B cells

**Fig. 3:**
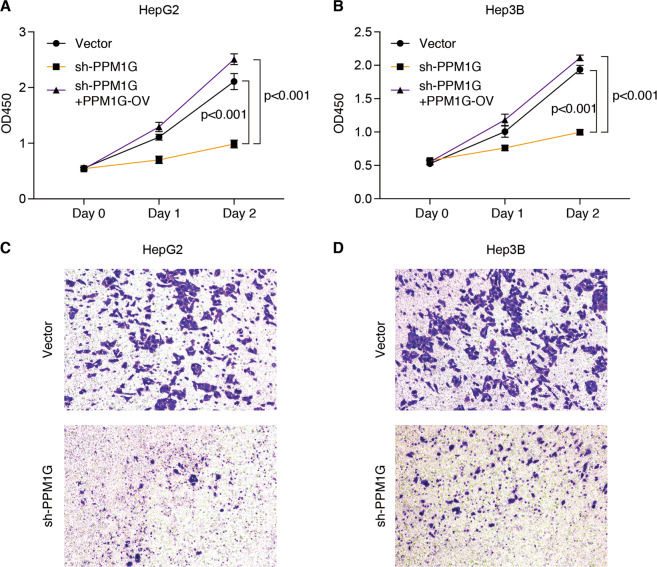
Knockdown PPM1G inhibited cell proliferation and invasion of HepG2 and Hep3B cells. **A**–**B** Knockdown PPM1G inhibited the cell growth of HepG2 and Hep3B. HepG2 and Hep3B cells were transfected with shRNA targeting the PPM1G, or co-transfected with PPM1G shRNA and PPM1G overexpression plasmid. Viable cells were examined at 0-, 1-, and 2-days post transfection by CCK-8 assays. **C**–**D** Knockdown PPM1G inhibited the invasion of HepG2 and Hep3B cells. HepG2 and Hep3B cells were transfected with shRNA targeting the PPM1G and the invasion was determined by transwell assays.

To explore the function of PPM1G in HCC, we analyzed the expression of PPM1G in hepatocellular cells. The expression of PPM1G was highly expressed in HCC cell lines as compared with human fetal hepatocyte line LO2 (Fig. S2). We then knock down the expression of PPM1G in HCC cell lines, including HepG2 and Hep3B cells. We found that the knockdown of PPM1G inhibited the growth of HepG2 and Hep3B (Fig. 3A–B). Meanwhile, the PPM1G knockdown resultant tumor growth suppression could be rescued by PPM1G overexpression (Fig. 3A–B). Next, we detected the effects of PPM1G in cell invasion by transwell assays. The invasion ability of HCC cells was inhibited after the knockdown of PPM1G, as the transwell assay showed that the knockdown of PPM1G decreased the transferred HepG2 and Hep3B cells (Fig. 3C–D). Together, these in vitro cell experiments indicated that a high level of PPM1G was required for cell growth and invasion.

### Knockdown PPM1G inhibited tumor growth in xenograft model of HCC

**Fig. 4:**
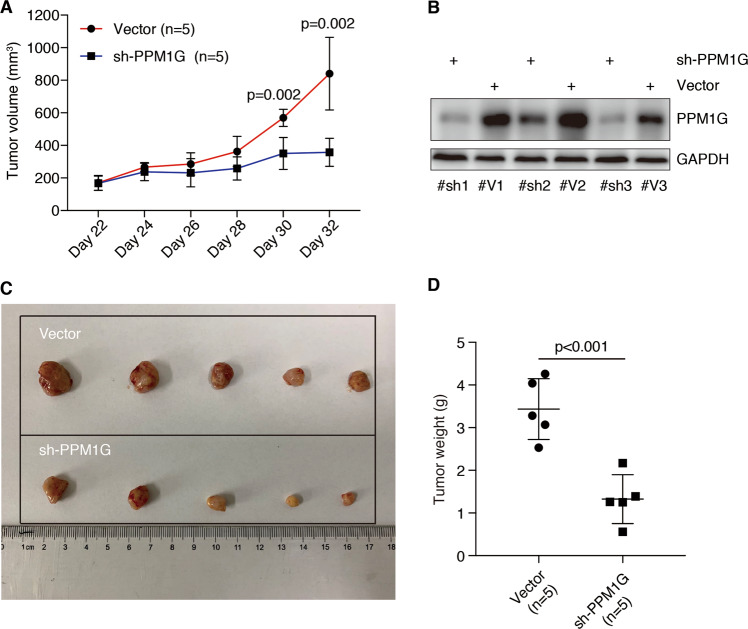
Knockdown PPM1G inhibited tumor growth of HCC cells in vivo. **A** PPM1G is required for tumor growth of Hep3B cells in nude mice. Hep3B cells with PPM1G knockdown or transfected with vector were injected into the nude mice (*n* = 5). Tumor sizes were examined after every two days after 22 days of transplantation. **B** Validate the expression of PPM1G in tumors. The expression of PPM1G in three pairs of transplanted tumors was examined. GAPDH was used as an internal control. **C** Decreased tumor size in HCC with PPM1G knockdown. Mice were sacrificed after 32 days of transplantation and the image of tumors was shown. **D** Decreased tumor weight in HCC with PPM1G knockdown. The tumor weight of each tumor was examined after 32 days of transplantation.

To further explore the PPM1G function in vivo, we applied a murine xenograft model for HCC. We subcutaneously transplanted the PPM1G knocked-down cells or vector-transfected cells into nude mice and observed the tumor volumes over time. As shown in Fig. 4A–C knockdown PPM1G significantly decreased the tumor volume. The tumor weight in the PPM1G knockdown group was also much less than that in the vector-transfected group (Fig. 4D). Together, these observations indicating knockdown PPM1G inhibited the tumor growth of HCC in vivo.

### PPM1G interacted with SRSF3 and dephosphorylated SRSF3

**Fig. 5:**
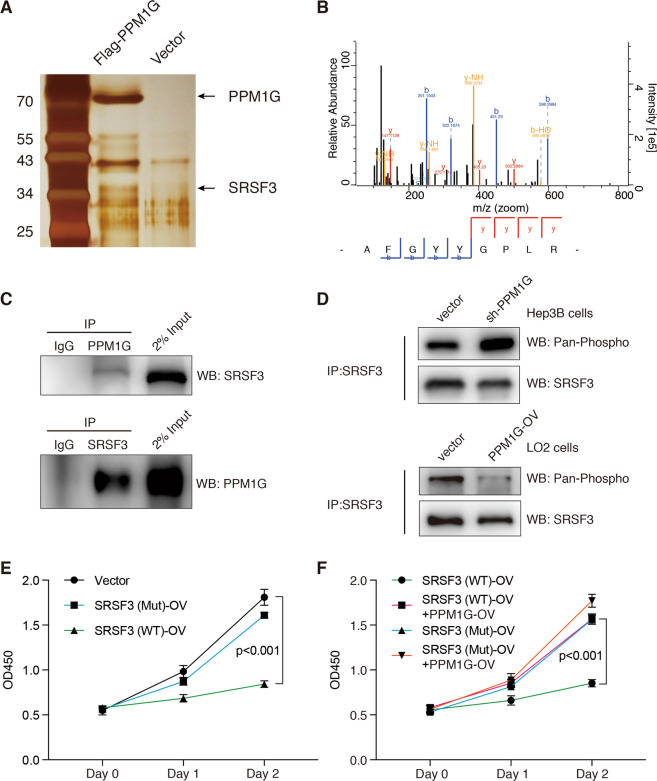
PPM1G interacts with SRSF3 in Hep3B cells. **A**–**B** Identify PPM1G-interacted proteins by immunoprecipitation of Flag-tagged PPM1G protein followed with LC-MS/MS. The silver staining of Flag-PPM1G immunoprecipitated proteins was shown (**A**). Representative peptides of the SRSF3 protein were illustrated (**B**). **C** Co-immunoprecipitation showed direct interaction between PPM1G and SRSF3. Antibodies against PPM1G or SRSF3 were used for immunoprecipitation. IgG was used as the negative control. About 2% of input proteins were loaded as an internal control. **D** PPM1G dephosphorylated the SRSF3. Hep3B cells were transfected with shRNA-targeting PPM1G or vector (upper panel). LO2 cells were transfected with PPM1G overexpression plasmid or vector (lower panel). All SRSF3 proteins were first immunoprecipitated using antibodies against SRSF3. The phosphorylated levels of SRSF3 were then detected using antibodies against pan-phosphorylated proteins. **E** Overexpression of SRSF3-WT inhibited Hep3B cell growth. Hep3B cells were transfected with SRSF3-WT, SRSF3-Mut, or vector plasmid. **F** PPM1G overexpression abolished SRSF3-WT-induced cell growth inhibition. Hep3B cells were co-transfected with SRSF3-WT/SRSF3-Mut and PPM1G overexpression plasmid. Viable cells were examined at 0-, 1-, and 2-days post transfection by CCK-8 assays.

Next, we analyzed PPM1G’s cellular function in HCC cells. We first performed PPM1G immunoprecipitation (IP) following a proteomic analysis and found that PPM1G interacts with proteins involved in RNA binding, including SRSF3 (Fig. 5A–B). Further co-IP experiments showed that PPM1G interacts with SRSF3, a protein related to alternative splicing (Fig. 5C). To further explore PPM1G’s function on the SRSF3 protein, we determined SRSF3 phosphorylation in HCC with or without PPM1G knockdown. We found that SRSF3 phosphorylation significantly increased in Hep3B cells with PPM1G knockdown (Fig. 5D). Meanwhile, we also found the PPM1G overexpression in LO2 cells resulted in decreased phosphorylation of SRSF3 protein (Fig. 5D). Thus, PPM1G might regulate the SRSF3 phosphorylation.

In addition, we overexpressed SRSF3, including the full-length SRSF3 (SRSF3-WT) and the SRSF3 with a deletion of SR enriched C-terminal region (SRSF3-Mut), in Hep3B cells. The SRSF3-WT, but not SRSF3-Mut, significantly suppressed the cell growth of Hep3B cells (Fig. 5E). Moreover, we co-expressed SRSF3 (SRSF3-WT or SRSF3-Mut) and PPM1G in Hep3B cells. The PPM1G overexpression significantly abolished the SRSF3-WT induced cell growth inhibition (Fig. 5F) but showed fewer impacts on SRSF3-Mut overexpressed cells. These observations indicating that SRSF3′s cellular function is largely controlled by PPM1G in HCC.

### PPM1G regulated the alternative splicing of genes involved in the cell cycle and transcriptional regulation

**Fig. 6:**
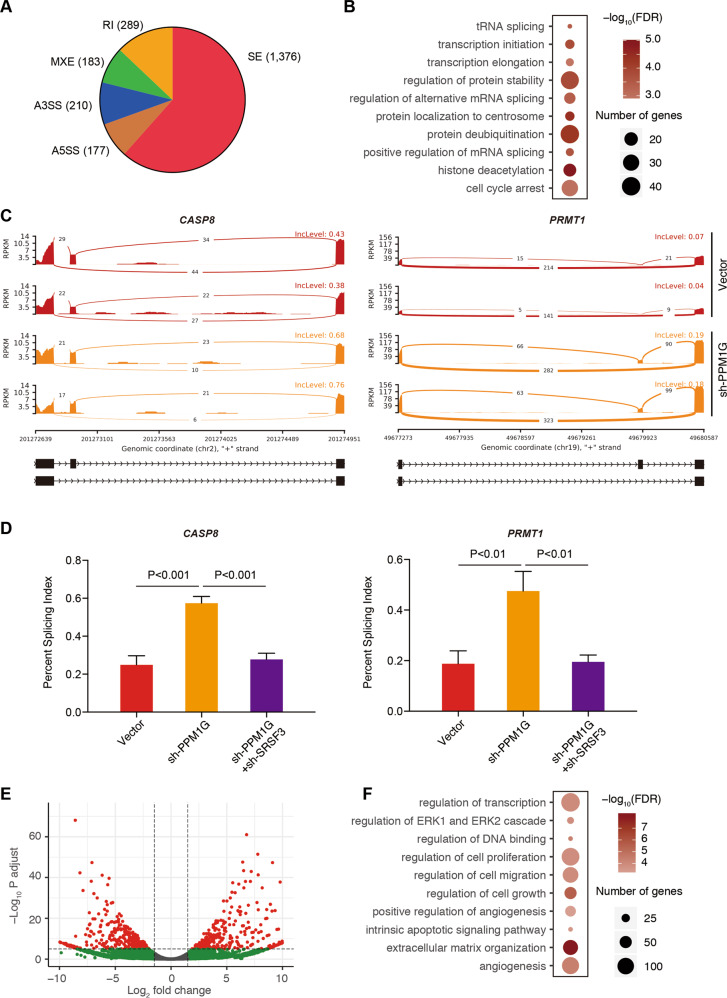
PPM1G regulated the alternative splicing in HCC. **A**–**B** Identify PPM1G-related alternative splicing events. Alternative splicing analysis was conducted in Hep3B cells with and without PPM1G knockdown. The pie plot shows the distribution of the alternative splicing events (**A**). A gene ontology analysis of differentially alternative splicing events in Hep3B cells with PPM1G knockdown was completed (**B**). **C** This illustration represents alternative splicing events of the CASP8 gene and PRMT1 gene. The reads at the junction site between two exons were plotted. The red color represents the RNA-seq of vector-transduced Hep3B cells, and the orange color represents the RNA-seq of sh-PPM1G-transduced Hep3B cells. **D** PPM1G’s alternative splicing regulation was achieved by SRSF3. The alternative splicing at the CASP8 and PRMT1 in Hep3B cells transfected with shRNA targeting the PPM1G, PPM1G + SRSR3, or vector were examined by real-time RT-qPCR. **E**–**F** The differential expressed genes (DEG) post-PPM1G knockdown were identified. DEGs were obtained by RNA-seq analyses of cells transfected with shRNA-targeting PPM1G or vector. The volcano plot was shown (**E**). Gene ontology analyses of the DEGs were plotted (**F**).

As the regulation of the phosphorylation/dephosphorylation of alternative splicing protein determined the splicing activities, we thus performed an RNA-seq analysis in Hep3B cells with or without PPM1G knockdown. We also analyzed the changes on alternative splicing events. A total of 2235 differential alternative splicing events (false discovery rate < 0.05) corresponding to 1382 genes have been identified, including 1376 skipped exon (SE), 177 alternative 5′ splice site (A5SS), 210 alternative 3′ splice site (A3SS), 183 mutually exclusive exons, and 289 retained intron (Fig. 6A and Table S1). The mRNA splicing of genes related to transcription regulation (such as *SIRT6*, *HDAC6*, and *PRMT1*), cell cycle arrest (such as *CASP8*, *CDK7*, and *CNOT2*), and RNA splicing (*DDX17*, *DDX5*, *HNRNPA1*) was enriched (Fig. 6B–C and Table S2).

To explore if the alternative splicing regulation was achieved by its effects on SRSF3, we analyzed the alternative splicing in PPM1G knockdown cells, PPM1G, and SRSF3 knockdown cells, and vector-transduced cells. Similar to the RNA-seq, the splicing of *CASP8* and *PRMT1* was altered after PPM1G knockdown (Fig. 6D). However, the effects of PPM1G on *CASP8* and *PRMT1* RNA splicing were abolished with SRSF3 knockdown (Fig. 6D), suggesting that the PPM1G regulated the alternative splicing via SRSF3.

Considering that the PPM1G regulated the alternative splicing of genes involved in multiple important biological processes, we further analyzed the differential expressed genes (DEGs). A total of 1922 DEGs were identified (Fig. 6E and Table S3). Gene ontology analysis showed genes related to cell growth, cell proliferation, and cell migration (Fig. 6F and Table S4), which agreed with the observed inhibition of cell growth and cell metastasis in HCC cells after PPM1G knockdown (Fig. 3).

### PPM1G promoter was regulated by multiple transcription factors and co-activators

**Fig. 7:**
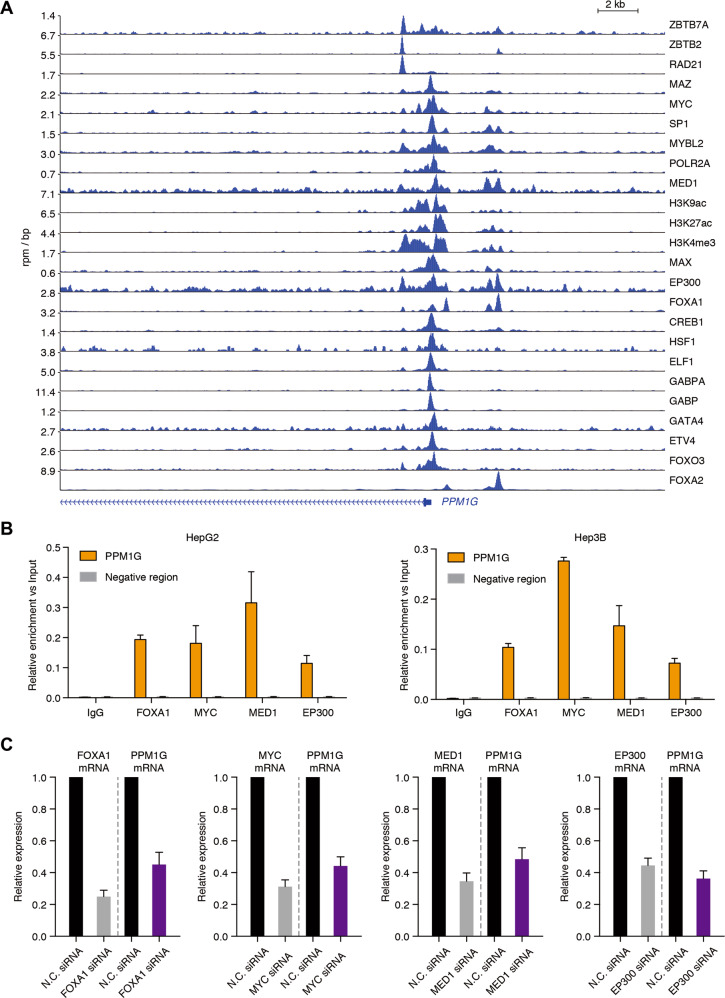
PPM1G was transactivated by multiple transcription factors and co-activators. **A** ChIP-seq analysis of transcription factors and co-activators binding on the regulatory region of PPM1G was completed. The ChIP-seq in HCC cell lines was retrieved from the GEO database. **B** ChIP-qPCR analysis of FOXA1, MYC, MED1, and EP300 binding on the promoter region of PPM1G in HepG2 and Hep3B cells was completed. **C** Knockdown of FOXA1, MYC, MED1, and EP300 decreased the expression of PPM1G. siRNA targeting FOXA1, MYC, MED1, EP300, or non-targeting siRNAs were transduced into the Hep3B cells. The knockdown efficiency and the expression of PPM1G were detected by RT-qPCR experiments.

Still, the mechanism underlying PPM1G overexpression in HCC remains unanswered. To address this issue, we collected ChIP-seq data in HCC cells and observed enrichment of H3K9ac, H3K27ac, and H3K4me3 signals at the promoter regions of PPM1G (Fig. 7A), suggesting that the promoter of PPM1G was highly activated in HCC cells.

To further illustrate how the promoter was activated, we analyzed the binding of multiple transcription factors and co-activators on the promoter regions of PPM1G. Multiple transcription factors, ZBTB7A, ZBTB2, MYC, MAZ, SP1, MYBL2, MAX, FOXA1, FOXA2, CREB1, HSF1, ELF1, GABPA, GABP, GATA4, ETV4, and FOXO3, were binding at the promoter regions of PPM1G (Fig. 7A). Meanwhile, the co-activators, including EP300, RAD21, and MED1, were also binding at the promoter regions of PPM1G (Fig. 7A). Together, these observations indicated that the PPM1G was highly transcriptionally activated in HCC cells.

To validate the multiple factor binding, we performed ChIP-qPCR experiments in HepG2 and Hep3B cells using antibodies against FOXA1, MYC, MED1, and EP300. We found all four of these proteins strongly bound at the promoter regions in HepG2 and Hep3B cells (Fig. 7B). In addition, we used siRNA to knock down the expression of the FOXA1, MYC, MED1, and EP300 in Hep3B cells and observed the transcription of PPM1G after the knockdown of these genes. Knockdown of FOXA1, MYC, MED1, or EP300 significantly decreased the levels of PPM1G (Fig. 7C), indicating that PPM1G was transactivated by the four transcription factors or co-activators in Hep3B cells.

## Discussion

Emerging evidence has shown that alternative splicing has a vital role in gene regulation, but the mechanism underlying the activation/deactivation of the alternative splicing machinery in malignancies remains largely unexplored. We here report that PPM1G, a phosphatase of splicing factor SRSF3, is aberrantly overexpressed in HCC. The PPM1G directly interacted with SRSF3 and dephosphorylated the SRSF3 in HCC. The dephosphorylated SRSF3 was released from the RNAs and lost its capacity to regulate alternative splicing, which led to exon inclusion/exclusion of its targets, such as *PRTM1* and *CASP8*.

PPM1G, a type of 2 C Ser/Thr phosphatase, has been reported to be the dephosphorylating regulator for many key proteins and is involved in many biological processes, such as transcriptional regulation, translational regulation, cell cycle regulation, and alternative splicing. It contributes to the sustained transcriptional activation activity of NF-κB by regulating the activity of 7SK snRNP and P-TEFb in transcription elongation [20, 21]. Studies have also reported the PPM1G involved in the dephosphorylation of histones H2A and H2B [22]. PPM1G is also involved in dephosphorylating the translation initiator 4E-BP1, thereby inhibiting the translation of its targets [23], such as Id1 in glioblastomas [24]. Recently, studies in innate immune response have shown that PPM1G can regulate immunosuppression by dephosphorylating the phosphorylate-STING and phosphorylate-MAVS proteins, which are key regulators of type I interferons during infections [25]. A previous study reported that PPM1G is physically related to the spliceosome [19], highlighting a potential function of PPM1G in alternative splicing. Indeed, a previous study showed that the PPM1G regulates the fourth and fifth exon inclusion of the CD44 gene via its interaction with YB-1 [18]. We reported that the PPM1G changed the alternative splicing of genes related to cell cycle arrest and transcriptional regulation, affecting gene expression related to cell proliferation, cell migration, and angiogenesis. Mechanistically, the PPM1G-mediated alternative splicing regulation was achieved by its regulation on the phosphorylation of SRSF3.

SRSF3 is an essential factor involved in the differentiation of normal hepatocytes, liver fibrosis suppression, and a potential suppressor for liver cancer tumors [26, 27]. It regulates the mRNA splicing of genes important for glucose and lipid metabolism, such as *Hnf1a*, *Ern1*, and *Dhcr7* in hepatocytes [26]. Knockout Srsf3 in mice impairs the maturation of the hepatocytes and drives spontaneous HCC with aging [26, 27]. Loss of Srsf3 induces aberrant splicing of genes related to EMT (epithelial to mesenchymal transition) and activates Wnt signaling and Myc activities [27]. Here, we found that the PPM1G regulated SRSF3, and high levels of PPM1G decreased SRSF3 activity in HCC cells. These observations support the notion that a high PPM1G level is an oncogenic event in HCC, which may occur via its negative regulation of SRSF3 activity.

By taking advantage of high-throughput sequencing technologies, many alternative splicing events have been intensively reported. We have reported the many alternative splicing events; among these, some key genes related to cell cycle and transcriptional regulation were regulated by PPM1G. We found that only about half of the sixth exon in *CASP8*, a key regulator for apoptosis and cell growth, was included under the steady status of the Hep3B cells. The inclusion was significantly increased after the PPM1G knockdown. Similarly, only a few co-activator *PRMT1* transcriptions include the 7th exon, but the inclusion level was significantly increased after the PPM1G knockdown. In addition, we demonstrated that the exon inclusion of *CASP8* and *PRMT1* relied on the SRSF3 protein. Although the function of the exon inclusion or exclusion isoforms of *CASP8* or *PRMT1* was not investigated in the current work, the effects of PPM1G in alternative splicing and the clinical impact, functional importance of PPM1G in HCC highlighted the essential role of PPM1G-mediated alternative splicing during the progression of HCC.

In summary, the PPM1G was activated by multiple transcription factors and co-activators, resulting in the abundant expression of PPM1G in HCC cells. The PPM1G interacted with the SRSF3 RNA splicing regulator and dephosphorylated the SRSF3, which led to the detachment of SRSF3 from RNA. The high levels of PPM1G negatively regulated the exon inclusion of CASP8, PRMT1, and many other genes, thereby promoting cell proliferation and metastasis of the HCC cells.

## Materials and methods

### Enrolled patients

This study was approved by the Ethics Committee of Jiangyin People’s Hospital, School of Medicine, Southeast University, and was conducted according to the Declaration of Helsinki. A total of 128 HCC patients in Jiangyin People’s Hospital were enrolled in this study. Written consent was acquired from all participants.

### Tissue microarray (TMA) and IHC

The TMA used in this study was conducted as previously described [28, 29]. Antibodies against the PPM1G (#15532-1-AP, Proteintech, Rosemont, IL, USA) were used for IHC staining. The ratio of the PPM1G-positive cells was scored from 1 to 4, and the levels of PPM1G in cells were scored from 0 to 4. The overall PPM1G score was obtained by multiplying the PPM1G-positive ratio score and the PPM1G-positive level score in cells. The PPM1G scores were examined by two pathologists independently.

### RNA extraction and real-time quantitative PCR (RT-qPCR)

The RNA was extracted with the TRIZOL (#15596026, Invitrogen, USA) per the manufacturer’s instructions. The RT-qPCR was performed with AceQ Universal SYBR qPCR Master Mix (#Q511-02, Vazyme, China) according to the manufacturer’s instructions. The primers were listed as follows: PPM1G-F: 5′-CTGCTTCAGACTACCAAACTGG-3′, PPM1G-R: 5′-CTCTGTAGAAATGGCAAACCACA-3′; FOXA1-F: 5′-GCAATACTCGCCTTACGGCT-3′, FOXA1-R: 5′-TACACACCTTGGTAGTACGCC-3′; MYC-F: 5′-GGCTCCTGGCAAAAGGTCA-3′, MYC-R: 5′-CTGCGTAGTTGTGCTGATGT-3′; MED1-F: 5′-GAGGGCATCAACATTTGGTCA-3′, MED1-R: 5′-AGATGAGAGCCCAGTCCATTC-3′; EP300-F: 5′-AGCCAAGCGGCCTAAACTC-3′, EP300-R: 5′-TCACCACCATTGGTTAGTCCC-3′; GAPDH-F: 5′-GGAGCGAGATCCCTCCAAAAT-3′, GAPDH-R: 5′-GGCTGTTGTCATACTTCTCATGG-3′. Relative mRNA expression was calculated using the 2^−ΔΔCT^ comparative method.

### Analyze the expression of PPM1G using the GEO and TCGA database

For the PPM1G in the TCGA-LIHC cohorts, the expression of PPM1G was obtained from the UALCAN online analysis tool [30] (http://ualcan.path.uab.edu/analysis.html).

The expression of PPM1G was also retrieved from the microarray data sets in the GEO database (GSE102083 [31], GSE14520 [32], and GSE76297 [33]). The expression of PPM1G in normal liver samples, HCC samples, and adjacent normal tissues was plotted.

### Cell growth analysis and transwell assays

Cell growth was analyzed according to the manufacture’s instruction of the Cell Counting Kit-8 (CCK-8) (#CK04, Dojindo, Japan). In brief, the cells were seeded in a 96-well plate and transfected with plasmids for 4 h, and then the culture media were replaced and cultured for an additional 24 or 48 h. A 10 μL CCK-8 reagent was added to each well and incubated at 37 °C for 2 h. The absorbance at OD450 was detected by the Multiskan™ FC Microplate Photometer (Thermo Fisher). The transwell assays were conducted as previously reported [34]. The upper chamber was pre-treated with Matrigel matrix (#354230, BD, USA). Cells were seeded in the upper chamber in Dulbecco’s Modified Eagle Medium (DMEM) culture media without FBS. Ten percent FBS in DMEM was added into the lower chamber. After 24 or 48 h of culture, the migrated cells were detected by the crystal violet staining.

### Mice model

The murine studies were approved by the Animal Care and Use Committee of Jiangyin People’s Hospital, School of Medicine, Southeast University, and conducted according to the institutional protocols. Six-week-old BALB/c-nu/nu nude mice (purchased from Shanghai SLAC Laboratory Animal Co., Ltd., China) were used. The mice were randomly divided into two groups. Hep3B cells transfected with shRNA-targeting PPM1G or vectors were subcutaneously injected into the mice. The tumor volumes were assessed. All tumor burdened mice were sacrificed, and the tumor weight was evaluated.

### Western blot experiments

The western blot experiments were performed as previously described [35]. The antibodies were as follows: antibodies against the PPM1G were purchased from Proteintech (#15532-1-AP, Rosemont, IL, USA). Antibodies against the SRSF3 were purchased from the Santa Cruz Biotechnology (#sc-13510, Santa Cruz, CA, USA). Antibodies against the GAPDH were purchased from the Cell Signaling Technology (#2118, Beverly, MA, USA).

### RNA-seq and RNA-seq analysis

The RNA-seq experiments were performed with the VAHTS mRNA-seq V3 Library Prep Kit for Illumina (#NR611, Vazyme, China) according to the manufacturer’s instructions. The RNA-seq libraries were sequenced in the Illumina Nova-seq 6000 sequencer.

For the quantification of the transcript, the raw sequences were mapped to the reference genome (hg38) using the STAR2 algorithm [36]. The read counts of each gene were calculated using the HT-seq algorithm [37]. The DEGs were obtained by using the DEseq2 R package [38].

For the alternative splicing analysis, the alternative splicing events and the differential spliced events were identified by the rMATs algorithm [39]. The representative alternative splicing events were plotted by the ggsashimi tools [40].

For the functional annotation of the differential genes and the alternative splicing events, the topGO R package and the GSEA (gene set enrichment analysis) tools were used [41].

### Immunoprecipitation

The IP experiments were performed as previously described [42]. In brief, the cells were harvested and lysed in the IP dilution buffer (20 mM Tris-HCl (PH 8.0), 2 mM ethylenediaminetetraacetic acid (EDTA), 1% Triton X-100, 150 mM NaCl). The lysate was centrifuged, and the supernatant was collected for IP reaction. In all, 2.5 μg antibodies were added and incubated overnight at 4 °C. In brief, 25 μL protein G beads were added for further incubation for 4 h at 4 °C. The beads were washed four times with the IP wash buffer (20 mM Tris-HCl [PH 8.0], 2 mM EDTA, 1% Triton X-100, 350 mM NaCl), and the protein complexes were eluted with the IP elution buffer (25 mM Tris-HCl PH 7.5, 10 mM EDTA, 0.5% sodium dodecyl sulfate). The eluted samples were subjected to proteomic study or western blot analysis. The proteomic (LC-MS/MS) analysis was conducted in the Shanghai Dianxi Biotech company.

### Chromatin immunoprecipitation (ChIP)

For the ChIP-seq reanalysis, the ChIP-seq datasets were obtained from the cistrome database (http://cistrome.org/db/). The binding of multiple transcription factor/co-regulators among the regulatory regions for PPM1G was plotted in the WashU browser.

The ChIP experiments were performed with the ChIP-IT high sensitivity kit (#53040, Active Motif, Carlsbad, CA, USA) according to the manufacturer’s instructions. The antibodies against FOXA1 (#ab170933, Abcam), MYC (#9402, Cell Signaling Technology), MED1 (#17-10530, EMD Millipore, Temecula, CA, USA), and EP300 (#ab14984, Abcam) were used. The primers against the promoter regions and distal desert regions were listed as follows:

PPM1G promoter-F: 5′-GGGCAGTCATTTCCTTAGCA-3′; PPM1G promoter-R: 5′-GGGAACGAAGGAAGAGGTTC-3′; ChIP-negative-F:5′-CCCATCTTCTTTCCTGACCA-3′; ChIP-negative-R:5′-TCAGAGAAGCACCACCTCCT-3′. The qPCR was performed with the ChamQ SYBR Color qPCR Master Mix (Low ROX Premixed) (#Q431-02, Vazyme, China) in the Light Cycler® 480 Real-Time PCR System.

### Statistical analysis

Differential expression of PPM1G among different groups was examined by the Mann–Whitney *U* test. Analysis of cell/tumor growth was performed with the student’s *t* test. The significance of survival analysis was evaluated by the log-rank test. Statistical analysis was conducted with the GraphPad prism 7.0. All tests were performed with the two-sided method, and the statistical *p* value < 0.05 was considered significant in this study.

## Supplementary information

Supplementary Figures and Figure Legends

Table S1

Table S2

Table S3

Table S4
