# Supplementary material for: PPM1G promotes the progression of hepatocellular carcinoma via phosphorylation regulation of alternative splicing protein SRSF3
Source: Cell Death Dis. 2021 Jul 21;12(8):722. doi: 10.1038/s41419-021-04013-y (PMC8295330; doi:10.1038/s41419-021-04013-y)
Supplement: Supplementary file 1 — Supplementary Figures and Figure Legends [file 41419_2021_4013_MOESM1_ESM.docx]

**Supplementary Figures and Figure Legends**


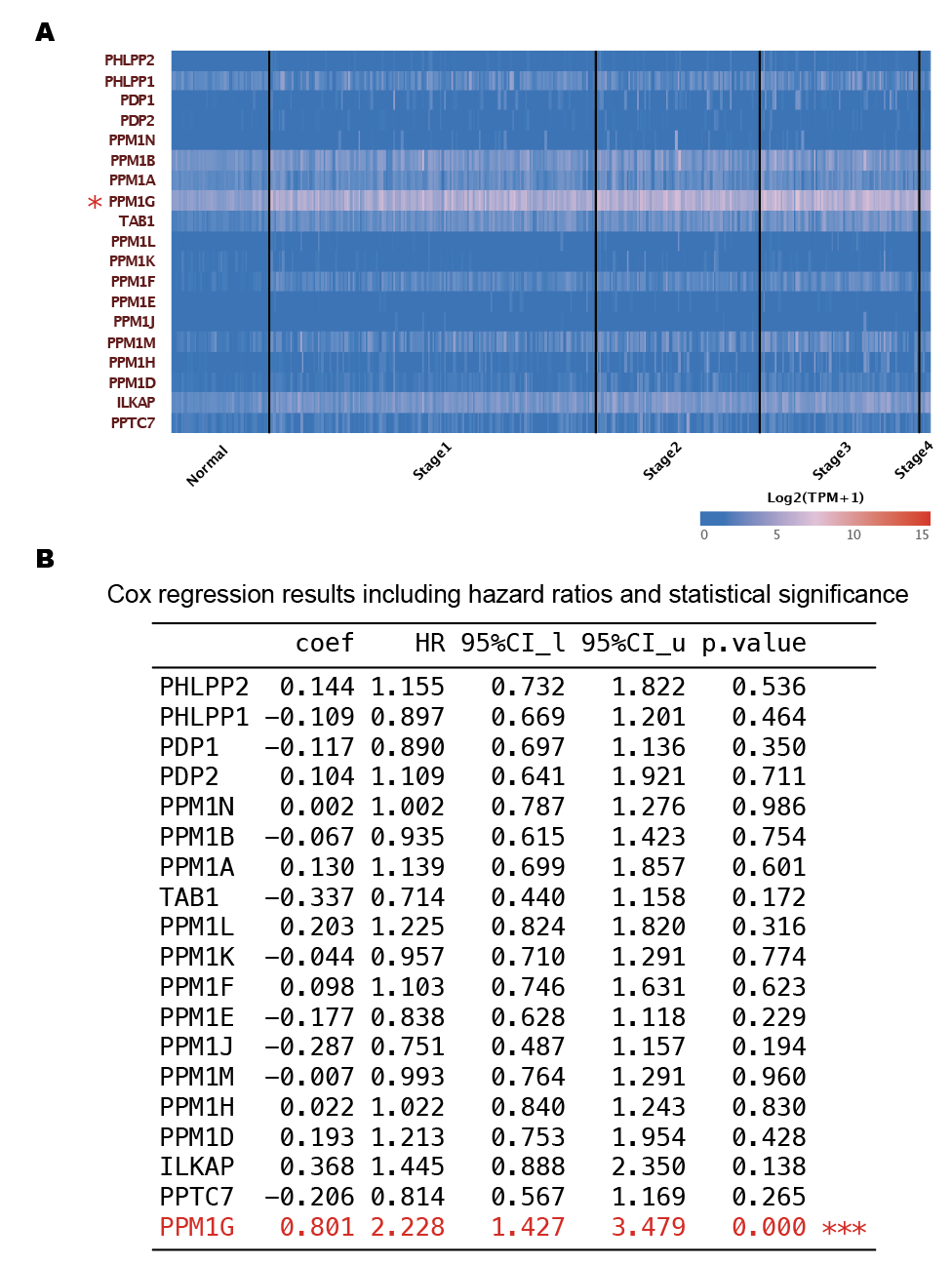


**Figure S1. Identify abnormally expressed protein phosphatases in hepatocellular carcinoma.**

(A) The expression of protein phosphatases in the normal liver tissues and four different stages of hepatocellular carcinoma. The mRNA expression was obtained from the TCGA-LIHC cohort. (B) Prognostic impact of the protein phosphatases in hepatocellular carcinoma. The hazard ratio of the protein phosphatases in hepatocellular carcinoma was shown. The hazard ratio was computed by the Log-rank test.


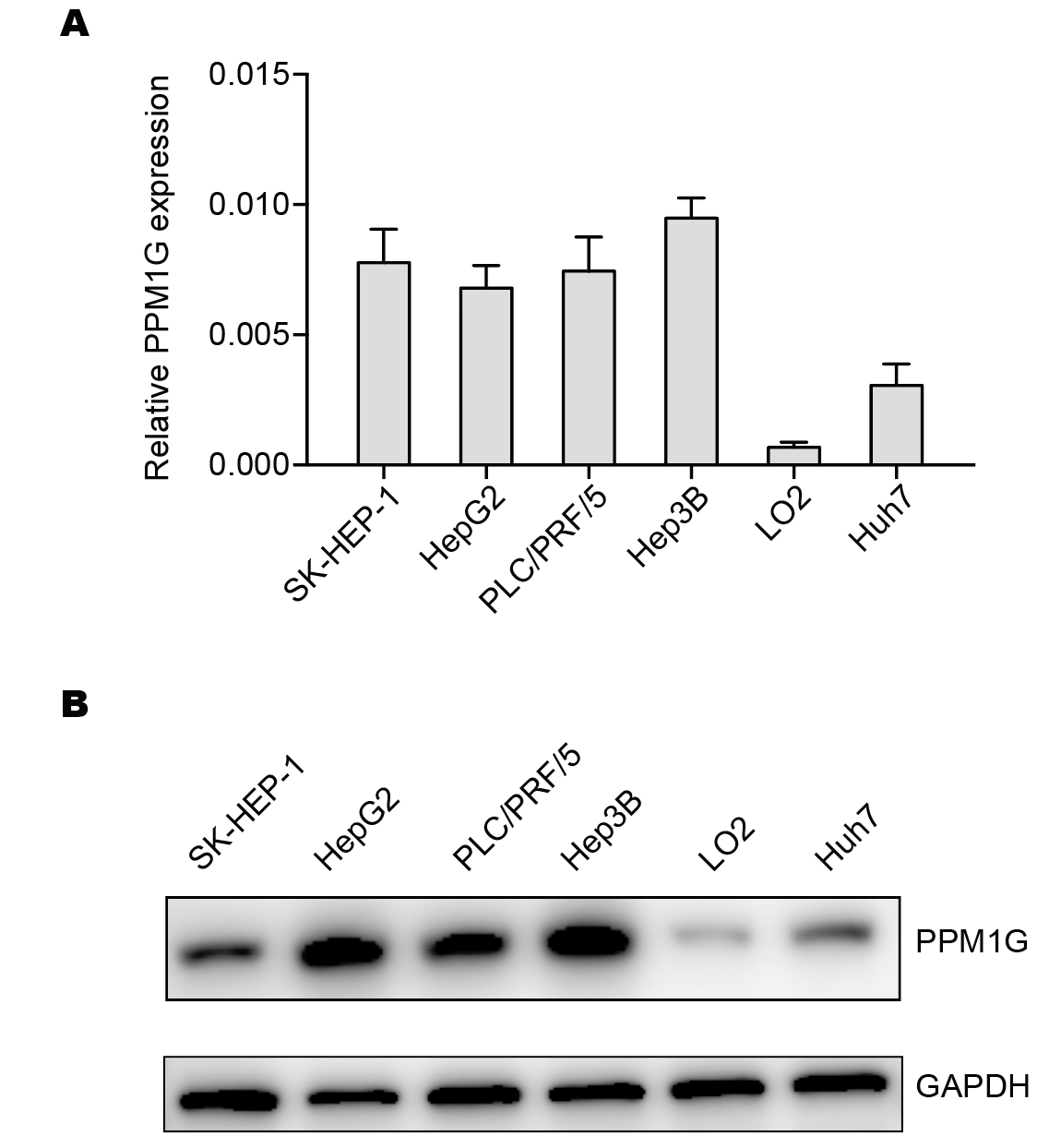


**Figure S2. Examine the expression of PPM1G in hepatocellular cells.**

(A) Relative mRNA expression of PPM1G in SK-HEP-1, HepG2, PLC/PRF/5, Hep3B, LO2, and Huh7 cells. The expression of PPM1G was normalized using the GAPDH. (B) The protein levels of PPM1G in SK-HEP-1, HepG2, PLC/PRF/5, Hep3B, LO2, and Huh7 cells. GAPDH was used as the internal control.
